# Autoantibodies in small fiber neuropathy: frequency and clinical features associated with antibodies to the novel targets MX1 and DBNL

**DOI:** 10.1007/s00415-026-13995-8

**Published:** 2026-07-17

**Authors:** Luana Morelli, Fortuna Ricciardiello, Alessandro Furia, Alex Incensi, Stefano Vozzi, Lucrezia Serra, Ilaria Gligora, Veria Vacchiano, Giovanni Rizzo, Vincenzo Donadio, Rocco Liguori, Maria Pia Giannoccaro

**Affiliations:** 1 Programma di Patologia Neuromuscolare E Neuroimmunologia, IRCCS Istituto Delle Scienze Neurologiche di Bologna, Bologna, Italy; 2 Dipartimento di Scienze Biomediche E Neuromotorie, Università di Bologna, Via Altura 3, 40139 Bologna, Italy; 3 UOC Clinica Neurologica, IRCCS Istituto Delle Scienze Neurologiche di Bologna, Bologna, Italy

**Keywords:** SFN, Autoantibody, MX1, DBNL, Pain, Autoimmunity, Idiopathic, Covid-19

## Abstract

Small fiber neuropathy (SFN) is characterized by neuropathic pain and autonomic dysfunction. Emerging evidence suggests a potential autoimmune component, although a limited number of autoantibody targets have been implicated so far. This study aimed to investigate the prevalence of antibodies against the intracellular targets MX1, DBNL, and KRT8 in SFN. In this prospective observational study, consecutive patients with suspected SFN underwent serum collection and skin biopsy. Sera were screened for MX1, DBNL, and KRT8 antibodies using fixed in-house cell-based assays (CBA). One hundred disease controls were included to confirm autoantibody specificity. Patients were classified by final diagnosis and etiology as idiopathic (iSFN) or secondary (sSFN). A separate replication cohort of 31 iSFN patients was tested to corroborate the main cohort findings. Antibody binding specificity was confirmed by co-localization and immunoadsorption assays. In the primary cohort, 4/258 patients (1.5%) tested positive: 2 for MX1-IgG, 2 for DBNL-IgG, none for KRT8-IgG. All seropositive patients showed reduced intraepidermal nerve fiber density consistent with SFN. MX1 cases exhibited somatic and autonomic iSFN; DBNL cases included both an autoimmune sSFN and iSFN with symptoms onset post-COVID-19-vaccination. In the replication cohort, one patient (3.2%) tested positive for MX1-IgG, while none were positive for DBNL-IgG. MX1 and DBNL antibodies were rare in SFN, and no KRT8 reactivity was detected. Although the intracellular localization of these antigens makes a direct pathogenic role unlikely, MX1-seropositive patients shared clinical features including older age, diffuse burning pain, and pruritus, suggesting MX1-IgG may serve as a biomarker of a distinct iSFN subtype.

**Supplementary Information:**

The online version contains supplementary material available at 10.1007/s00415-026-13995-8.

## Introduction

Small fiber neuropathy (SFN) is a disorder affecting thinly myelinated Aδ fibers and unmyelinated C fibers[1, 2], leading to sensory disturbances like burning pain, tingling, numbness, and pins and needles sensation. Autonomic symptoms are also common and may include dry eyes and mouth, cardiac arrhythmias, orthostatic hypotension, gastrointestinal dysmotility, urinary dysfunction, sexual disturbances, sweating abnormalities, and skin discoloration[2–4].

Although the etiology of SFN is heterogeneous, a growing body of evidence suggests an autoimmune pathogenesis in a subset of patients. This hypothesis is supported by some observations of patients’ response to immunotherapy and by the detection of autoantibodies in some cases [5–7]. However, few autoantibody targets, against intracellular antigen such as FGFR3[8] and AGO[9] or extracellular antigens such as TS-HDS [10] and Plexin-D1 [7, 11, 12], have been identified so far in variable frequencies, and their pathogenetic role remains uncertain in most cases[7].

Recently, Chan et al.[13], employed a high-throughput autoantibody screening platform (Sengenics Immunome Protein Array) to identify potential autoantibody targets in SFN. Among the putative antigens identified were interferon-induced GTP binding protein MX1 (MX1), drebrin-like protein (DBNL), and keratin type II cytoskeletal 8 (KRT8). A subgroup analysis within the main cohort suggested MX1 as a potential biomarker for idiopathic SFN (iSFN).

Subsequently, the same authors investigated the presence of MX1-Abs in the same cohort using different detection methods, observing higher Abs levels in SFN than in HCs and, in particular, in a subgroup of autoimmune SFN. Functional studies have supported their role in the pathophysiology of SFN [14].

However, their findings require validation in larger, independent cohorts.

In this study, we developed and applied an *in-house* fixed cell-based assay (CBA) to screen for autoantibodies against MX1, DBNL, and KRT8 in a large cohort of patients with suspected SFN. We evaluated the frequency of these antibodies, their clinical associations and diagnostic relevance, and validated the results in an independent iSFN cohort.

## Materials and methods

### Study design and patients

We recruited two cohorts of consecutive patients referred to the IRCCS Istituto delle Scienze Neurologiche di Bologna for suspected SFN. The first cohort was recruited between September 2020 and December 2022. Consecutive enrolment was ensured by including every eligible patient meeting predefined clinical and electrophysiological criteria during this period, regardless of etiology. Detailed clinical, neurophysiological, and laboratory data were collected prospectively at the time of evaluation.

Following preliminary analyses in this initial group, we retrospectively selected, among a second (confirmatory) cohort of patients prospectively enrolled between February 2024 and April 2024, only those diagnosed with idiopathic small fiber neuropathy to validate the observed findings in a more homogeneous patient population.

Exclusion criteria for both cohorts included clinical signs of large fiber involvement (e.g. impaired vibration or light touch, hyporeflexia, ataxia) and any abnormalities on nerve conduction studies (NCSs). All patients underwent neurological examination, skin biopsies from the leg and thigh, NCSs to exclude large-fiber neuropathy, and blood screening to investigate secondary causes of SFN, including complete blood count, renal and liver function panels, electrolyte profile, thyroid function tests (TSH and free T4), fasting glucose, hemoglobin A1c, vitamin B12, folate, autoimmune screening (antinuclear antibodies and extractable nuclear antigen panels, rheumatoid factor, erythrocyte sedimentation rate, C-reactive protein, complement levels -C3, C4-, angiotensin-converting enzyme levels, celiac serology, thyroid related antibodies), serum protein electrophoresis with immunofixation, infectious testing (HIV, hepatitis B and C serologies). Additional investigations, including oral glucose tolerance test or genetic testing (i.e. for amyloidosis, connective disorders, Fabry disease etc.), were performed when clinically appropriate. Patients’ selection is shown in supplementary Fig. 1 (S1).

SFN diagnosis was established according to the NEURODIAB Criteria [15] based on a grading as possible, probable, or definite, and Besta Criteria (i.e. sensory/sign symptoms of small fiber impairment such as pinprick and thermal sensory loss and/or allodynia and/or hyperalgesia together with reduced intraepidermal nerve fiber density (IENFD)[16]. Patients diagnosed with SFN were further subtyped according to  the distribution of epidermal nerve fiber loss (length-dependent *vs* non-length-dependent pattern), and etiology (idiopathic *vs* secondary SFN). Secondary SFN was defined by the presence of an identifiable underlying condition (e.g., metabolic, autoimmune, infectious or genetic), whereas idiopathic was defined by the absence of a detectable cause after comprehensive screening. Some patients complained of burning pain and/or autonomic symptoms after a COVID-19 infection or vaccination. For the latter cases, inclusion criteria were: (1) burning pain starting within 2 months from the COVID-19 infection or a vaccine against COVID-19 and persisting for at least 12 months; (2) interval between the vaccine and the COVID-19 infection of more than 6 months [17]. To minimize overestimation of causality, a conservative approach was adopted, particularly in cases with coexisting possible etiological factors or when patients were evaluated long after symptom onset, limiting the reliability of the temporal association. Accordingly, cases with identifiable comorbidities were classified as secondary SFN, whereas those without were considered idiopathic; the SARS-CoV-2 infection or vaccination was regarded as a trigger.

Patients not meeting the criteria for SFN were included as controls. A further control cohort, used to assess the specificity of the CBAs, included sera from 100 patients with non-inflammatory neurological disorders (NINDs), including neurodegenerative disorders (i.e. dementia, motor neuron disease (MND), infections, and functional neurological disorders, and other inflammatory neurological disorders (OINDs), i.e. multiple sclerosis (MS) and encephalitis. Patients without sensory symptoms were included in this cohort.

The study was approved by the local Human Ethics Committee (CE Indipendente AUSL di Bologna cod. CE 12073 and CE Area Vasta Emilia Centro cod. 566–2023-OSS-AUSLBO-23146) and followed the Helsinki Declaration for international clinical research involving human beings. All subjects gave their written informed consent for inclusion in the study.

### Serum samples

Peripheral blood was collected via venipuncture, and serum was separated by centrifugation at 2000 × g for 15 min at 4 °C. The supernatant was aliquoted into polypropylene tubes and stored at –80 °C until use. Hemolyzed, icteric, or lipemic samples were excluded from analysis.

### Skin biopsy

Three-millimeter punch biopsies were taken from hairy skin at two sites: the proximal thigh (15 cm above the patella) and the distal leg (10 cm above the lateral malleolus) of hairy skin. Skin samples were immediately fixed in cold Zamboni fixative and kept at 4 °C overnight. Fifty-micrometer-thick sections were obtained using a cryostat (Leica, CM1950).

For each patient, six free-floating skin sections (three from leg and three from thigh) were prepared from the skin biopsy and incubated overnight with a panel of primary antibodies, including rabbit pan-neuronal marker protein gene product (PGP) 9.5 (1:500; Abcam, ab108986), and mouse collagen IV (1:2000; Chemicon, Temecula, MAB1910) helping to identify and outline basal membrane during the IENFD counting and skin annexes such as sweat gland (SG) and arrector pili muscle (APM), during the quantification of autonomic innervation. After washes, sections were incubated overnight at 4°C with fluorophore-labeled secondary antibodies (1:400; mouse Alexa Flour 488 and rabbit cyanine 3; Jackson ImmunoResearch, 715–545-150 and 711–165-152, respectively).

Sections were analyzed under a fluorescent microscope (Zeiss, Axioskop 40). IENFD was quantified by counting only the epidermal fibers marked by PGP 9.5 crossings of the dermal–epidermal junction as previously described [18]. The normative values for IENFD were based on previously published data [19, 20]. Autonomic innervation density was quantified using the previously described automated technique known as the ‘unsharp mask filter’ which creates a composite image by subtracting the background color in the out-of-focus image from the base image expressing the autonomic innervation staining in target structures suc as SG and APM (Image Pro Plus, Media Cybernetics)[21].

### Cell-based assay and IgG isotypes

#### Fixed CBA for MX1, DBNL, and KRT8 antibody detection

HEK293T cells were transfected with full-length plasmids encoding for human MX1 (Origene, CAT#: RC227878), DBNL (Origene, CAT#: RC203435), or KRT8 (Origene, CAT#: RC209570). The green fluorescent protein (EGFP) was co-transfected to confirm transfection efficiency.

After 24 h, cells were fixed with cold 4% paraformaldehyde (PFA) for 10 min at room temperature (RT), permeabilized with 0.2% Triton X-100 + 1% BSA in PBS 1X for 5 min at RT, and blocked with 5% BSA in PBS1X for 1 h at RT.

Fixed cells were incubated with sera (1:40) or primary commercial antibody against each specific target or plasmid tag (1:50, MX1 Mouse Monoclonal Antibody, Origene, TA500357; 1:50, DBNL Rabbit anti-Human/Mouse/Rat Polyclonal, Proteintech, 13,015–1-AP; 1:2000, Anti-DDK (FLAG) monoclonal antibody, Origene, TA50011) diluted in blocking solution for 45 min at RT. Antibody binding was detected using appropriate secondary antibodies, including anti-human IgG Fcɣ (1:1000; Alexa Fluor^®^ 594 AffiniPure™ F(ab’)₂ Fragment Goat Anti-Human IgG, Fcγ fragment specific, Jackson ImmunoResearch, 109–586-008) or anti-rabbit and anti-mouse to primary commercial antibodies (1:1000, Cy™3 AffiniPure^®^ Donkey Anti-Rabbit IgG (H + L), Jackson Immunoresearch, 711–165-150; 1:1000, Cy™3 AffiniPure^®^ Donkey Anti-Mouse IgG (H + L), Jackson Immunoresearch, 715–165-152). In positive serum samples, subclasses were characterized using specific secondary antibodies against IgG1, IgG2, IgG3 and IgG4 (1:500; mouse anti-human IgG1 and mouse anti-human IgG4, mouse anti-human IgG3, Invitrogen, A10630, A10651, MH1031 respectively; 1:1000, mouse anti-human IgG2, ThermoFisher Scientific, 05–3500) followed by Cy3-conjugated anti-mouse secondary antibody (Cy™3 AffiniPure^®^ Donkey Anti-Mouse IgG (H + L), Jackson Immunoresearch, 715–165-150). Each assay included positive and negative controls.

The antibody binding to the expressed antigen was observed using a fluorescent microscope (Zeiss, Axioskop 40) and scored using a well-established visual scoring system from 0 (negative) to 4 (strong positive) based on the frequency and intensity of the observed signal [22]. Samples scoring ≥ 0.5 were retested; samples scoring ≥ 1 were considered as positive and further titrated using twofold serial dilutions to determine the endpoint titer, defined as the highest dilution still yielding a detectable signal (CBA score ≥ 1). The interpretation was performed by two operators (LM and MPG), blinded to the clinical data and to each other scores. After assigning scores separately, the two operators compared and commented on the results. In cases of disagreement, the sera were retested. Discrepancies were considered significant when one operator assigned a score ≥ 1 and the other < 1. In such cases, samples were retested and, if discordance persisted, jointly re-evaluated until consensus was reached.

Specificity was verified by testing seropositive sera on untransfected HEK cells under identical conditions.

#### Co-localization

To confirm target-specific binding in seropositive cases, co-localization assays were performed. Transfected HEK cells without EGFP were incubated with positive serum for one hour at RT. The serum IgG binding was visualized with incubation with an anti-human secondary antibody (1:1000, Alexa Fluor^®^ 488 AffiniPure™ Goat Anti-Human IgG (H + L), Jackson ImmunoResearch, 109–545-003) and then with the commercial primary antibody, revealed with a secondary antibody conjugated with Cy3 fluorophore to evaluate a possible overlap of signals. Controls included blank conditions with only blocking buffer and secondary antibodies to assess non-specific background.

#### Immunoadsorption

To evaluate binding specificity, serial immunoadsorption was performed by incubating diluted serum with cells, transferring the supernatant sequentially through multiple wells of transfected or untransfected cells, the latter used as control. The final supernatant was tested for residual antibody reactivity using the CBA as described above.

### Statistical analysis

Descriptive statistics were used to summarize patient characteristics. Categorical variables were presented as counts and percentages, while continuous variables were described using medians and interquartile ranges (IQR). Comparisons between groups were made using Fisher’s exact test or Chi Square test for categorical variables, as appropriate, and the Mann-Whitney U test for continuous variables. Comparisons across more than two groups were performed using the Kruskal-Wallis test. A p-value < 0.05 was considered statistically significant. Statistical analyses were performed using IBM SPSS statistics version 25.0 (SPSS Inc., Chicago, USA). Graphs were plotted with GraphPad Prism version 8 (GraphPad software, San Diego, California, USA).

## Results

### Patient cohorts characterization

**Fig. 1:**
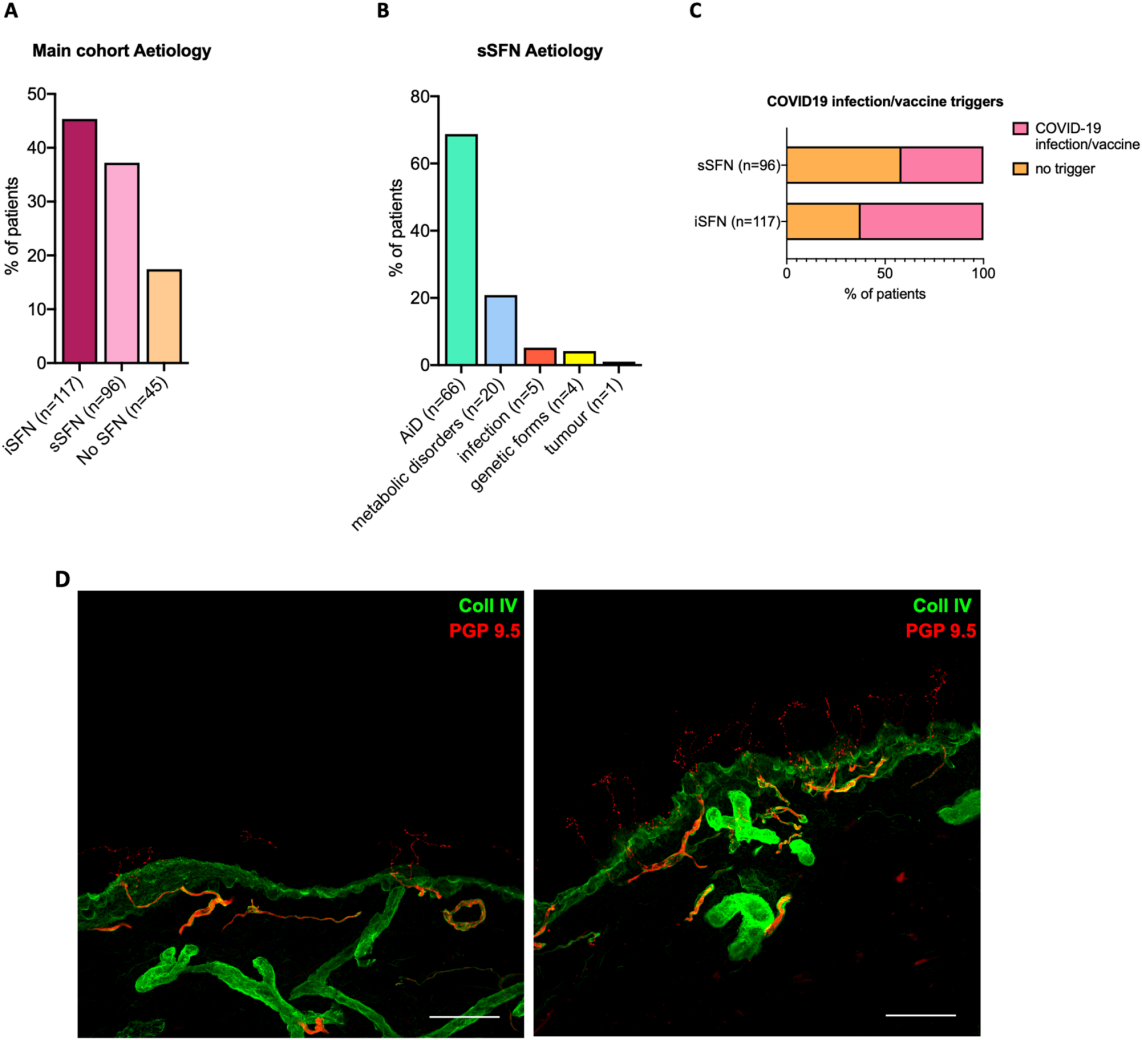
Aetiology distribution and characterisation of SFN cohort. **A** The distribution of patients from the main cohort by aetiology; **B** Percentage and distribution of different causes of sSFN in the main cohort; **C** Percentage of iSFN and sSFN patients in the main cohort that reported symptom onset following COVID-19 vaccination/infection; **D** Confocal images of immunostaining on skin sections showing the density of nerve fibers marked by PGP 9.5 (red) crossing the basal lamina at the dermal-epidermal border, labeled by Coll IV (green). In particular, the skin biopsy from a normal control (right panel) shows abundant, normal intraepidermal innervation, whereas a patient with SFN (left panel) exhibits reduced intraepidermal nerve fiber density. *AiD* autoimmune disease, *iSFN* idiopathic small fiber neuropathy, *noSFN* pain syndrome, *sSFN* secondary small fiber neuropathy

We included 258 consecutive patients (198 F, 60 M), with a median age of 47 years (IQR 18). Based on clinical, electrophysiological, and skin biopsy findings, 213 (82.56%) patients were diagnosed with SFN, whereas ten patients were diagnosed as having ‘probable’ SFN based on the NEURODIAB criteria, because quantitative sensory testing (QST) was unavailable. In particular, 117 (45.3%) cases were considered idiopathic, and 96 (37.2%) secondary (sSFN). The remaining 45 patients (17.4%), without a small nerve fiber pathology, served as disease controls (Figs. 1A, 1D). These patients were referred for suspected small fiber neuropathy but did not exhibit neuropathic pain characteristics, as they lacked allodynia and hyperalgesia, and displayed normal epidermal innervation. Their symptoms included isolated paraesthesia (11%), pain (62%), often diffuse (38.8%), burning (53%),constrictive/muscular (26.6%) or articular (31.1%), triggered or worsened by COVID-19 infection or vaccination in over half of cases. All patients had a normal examination and blood testing excluded conditions predisposing to SFN. In a few patients (13.3%) symptoms were transitory and resolved spontaneously. Others received a diagnosis of fibromyalgia (26.6%); in the remaining cases, pain was correlated to other medical conditions.

No significant age differences were observed between patients with and without SFN; a higher proportion of male patients was observed among those with iSFN (Table 1). Secondary SFN was mainly attributed to autoimmune disease or metabolic disorders (including B12 deficiency and diabetes) (Fig. 1B). Among patients with SFN, 13 (6.1%) had a history of malignancy (breast, n = 4; uterine, n = 2; lymphoma, n = 2; thyroid, n = 2; prostate, n = 1; kidney, n = 1; melanoma, n = 1), with cancer diagnosis preceding the onset of sensory symptoms by a median of 10 years (range 2–29 years). Additionally, six patients had low-grade or benign tumours. None of these cases was considered paraneoplastic, based on the temporal relationship, clinical features, tumour type, and negative onconeural antibody testing. Within the iSFN group, 55 patients (47%) reported symptom onset following COVID-19 vaccination, and 18 (15.4%) following COVID-19 infection. Within the sSFN group, 30 patients (31.3%) reported post-vaccine symptom onset, and 10 (10.4%) reported symptoms following COVID-19 infection (Fig. 1C).

**Table 1:** Characterization of Control, SFN and independent replication cohorts

	SFN Cohort				Independent replication cohort		Control Cohort
*Diagnosis*	*iSFN*	*sSFN*	*No SFP*	*P value*	*iSFN*	*P value*	
N. of subjects (%)	117 (45.3)	96 (37.2)	45 (17.4)		31 (100)		100 (100)
Gender							
Male (%)	38 (32.5)	17 (17.7)	5 (11.1)	0.004	11 (35.5)	ns	54 (54)
Female (%)	79 (67.5)	79 (82.3)	40 (88.9)		20 (64.5)		46 (46)
Median age (IQR)	45 (18)	49 (16)	44 (17.5)	ns	47 (20.5)	ns	64 (26.5)
Median age at onset (IQR)	41.5 (19.5)	45 (19.75)	41 (18)	ns	39 (19)	ns	na
Mean IEFNDs ± SD at leg	6.75 ± 3.07	6.46 ± 2.77	13.4 ± 2.5	< 0.0001	5.98 ± 2.25	ns	na
Mean IEFNDs ± SD at thigh	9.26 ± 3.42	8.47 ± 3.35	16.82 ± 2.72	< 0.0001	8.51 ± 2.35	ns	na
Pattern							
LD (%)	16 (13.7)	14 (14.6)	na	ns	5 (16.1)	ns	na
NLD (%)	93 (79.5)	80 (83.3)	na	ns	26 (83.9)		na
Onset after Covid 19 vaccine (%)	60 (51.3)	37 (38.5)	21 (46.7)	ns	0	< 0.0001*	0
Onset after Covid 19 infection (%)	24 (20.5)	17 (17.7)	11 (24.4)	ns	0	0.02*	0

*iSFN*   idiopathic small fiber neuropathy, *LD*   length-dependent, *M*   Male, *na*   not available, *ns*   not significant, *NLD*   non-length-dependent pattern, *sSFN*   secondary small fiber neuropathy, *SFP*   small fiber pathology, *IEFNDs*   Intraepidermal fiber nerve densities

A separate independent replication cohort included 31 additional iSFN patients (20 F, 11 M; median age: 47 years, IQR: 20.5). Only one case in this cohort had a history of breast cancer 18 years before symptoms onset. No significant differences were observed between the demographic and clinical features of idiopathic patients in the two cohorts (Table 1).

Finally, we included a NINDs control cohort of 100 patients (54 F, 46 M), with a median age of 64 years (IQR: 26.5) (Table 1), significantly older compared to patients included in the other cohorts (p < 0.001, Kruskal-Wallis test).

### Fixed CBA results in SFN and control cohorts

**Fig. 2:**
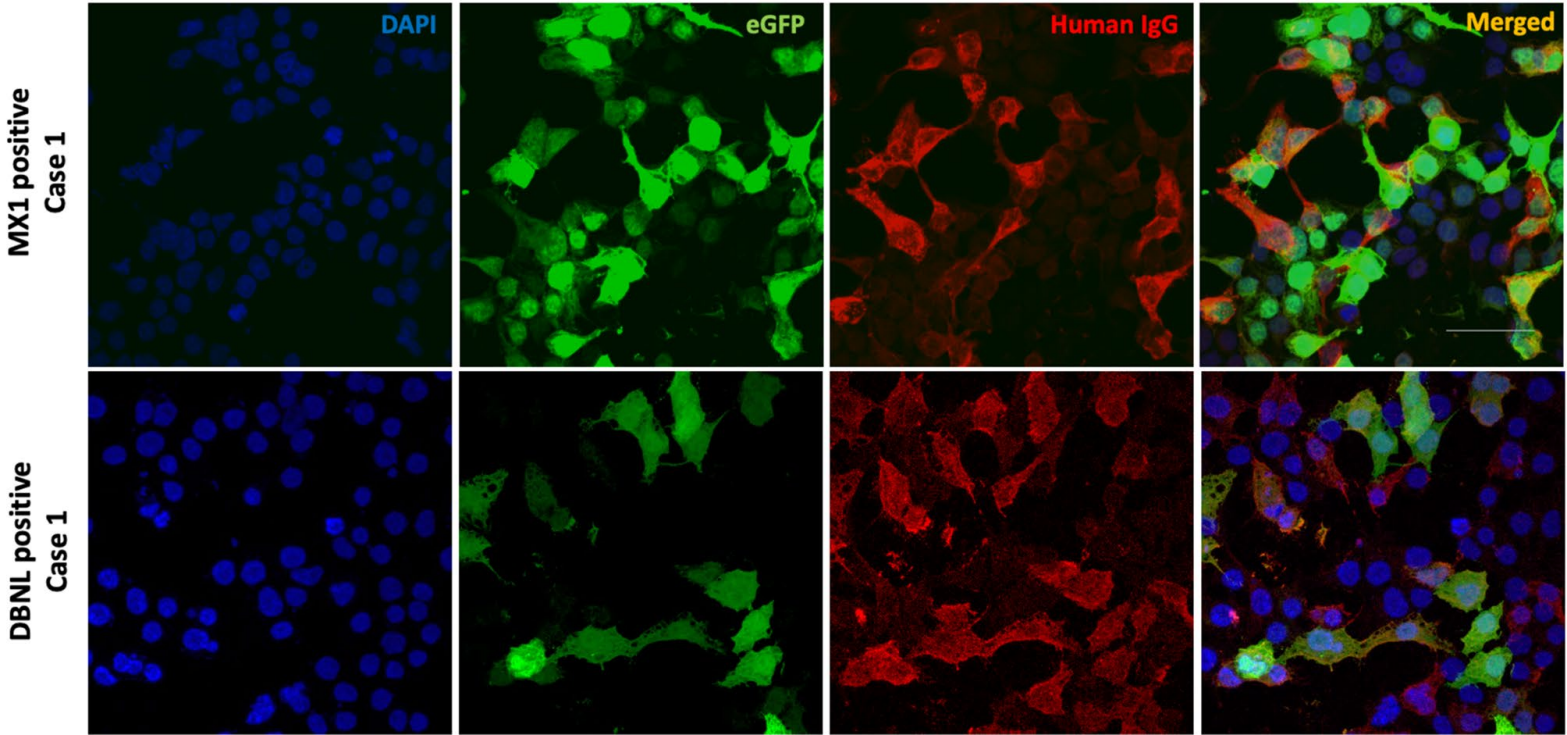
Positive cases on cell-based assays (CBAs). Confocal microscopy images showing serum immunostaining of patients with SFN on HEK293T cells expressing the MX1 or DBNL targets. Transfected cells are visualized in green (MX1 or DBNL expression); binding of IgG from positive patient is detected using an anti-human IgG antibody (red); nuclei are visualized with DAPI (blue). The representative image shown from a MX1-IgG positive case depicts a strong positivity, while DBNL-IgG staining shows a clear, intermediate-positive signal. Merged images show the co-localization between the red and green signal indicating the specificity of the binding.

In the main cohort, 4 of 258 sera (1.5%) tested positive using fixed cell-based assays (CBAs): 2 for MX1-IgG and 2 for DBNL-IgG (Fig. 2). No reactivity against KRT8 was observed. Among SFN patients, seropositivity was identified in 4 of 213 (1.9%), including 3 of 117 iSFN cases (2.6%) and 1 of 96 sSFN cases (1.04%). None of the control subjects tested positive.

When stratified by putative triggers, autoantibodies were found in 2 of 44 (4.5%) iSFN cases without known triggers and 1 of 55 (1.8%) with post-COVID-19 vaccination onset. All seropositive individuals but one exhibited a non-length-dependent (NLD) pattern of intraepidermal nerve fiber loss.

In the independent replication cohort, one additional case (3.2%) tested positive for MX1-IgG, while no DBNL-IgG antibodies were detected.

None of the seropositive cases had a history of cancer or developed malignancy after symptom onset, except for one MX1-IgG-positive patient who had a history of breast cancer 18 years prior to onset, with no evidence of recurrence at the time of evaluation.

Clinical features of positive and negative cases are summarized in Table 2.

**Table 2:** Summary of main features of seronegative and seropositive SFN cases

	SFN cases (seronegative) (n = 239)	MX1-IgG positive (n = 3)	DBNL-IgG positive (n = 2)
Acute onset (%)	28 (11.5)	1 (33.3)	1 (50)
Episodic worsening (%)	53 (22.1)	2 (66.6)	0
Paresthesia (%)	200 (83.7)	3 (100)	1 (50
Burning pain (%)	153 (64)	3 (100)	1 (50)
Itch (%)	22 (9.2)	2 (66.6)	0
Autonomic Symptoms (%)	58	0	1 (50)
*SFN Pattern*			
LD (%)	34 (14.2)	1 (33.3)	0
NLD (%)	195 (81.6)	2 (66.6)	2 (100)
* SFN type*			
Somatic	159 (66.5)	2 (66.6)	0
Autonomic	0	0	0
Mixed	40 (16.7)	1 (33.3)	2 (100)

*LD* length-dependent, *NLD* non-length-dependent

### Confirmation assays

**Fig. 3:**
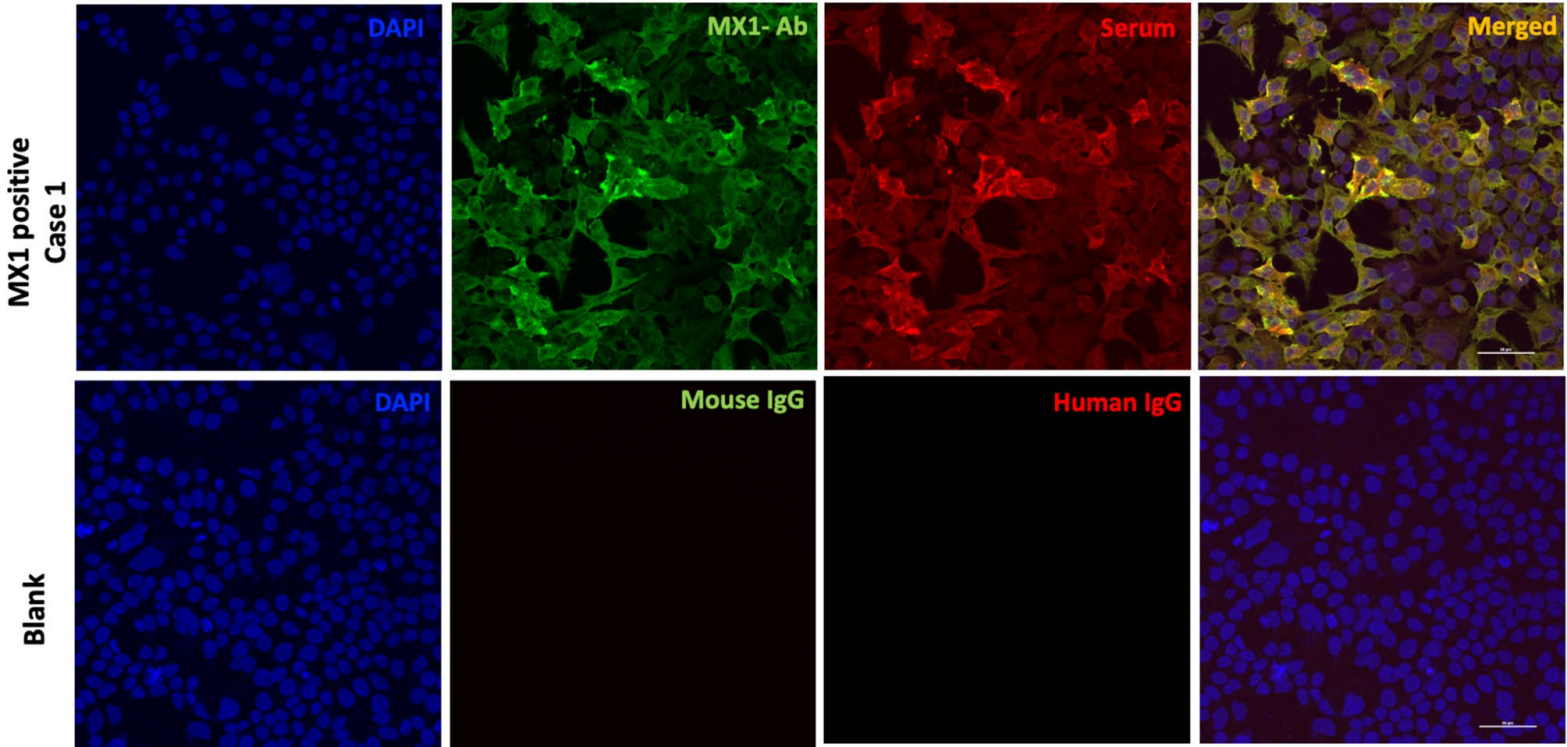
Co-localization assay. Confocal microscopy images showing a representative co-localization assay using a strong positive serum. Nuclei were visualized with DAPI (blue); HEK293T cells, transfected to express MX1, were incubated with a commercial MX1-Ab (green) and with iSFN patient serum (red); the merged image demonstrates co-localization of patient serum with MX1 staining. A blank control is included for comparison to highlight the specificity of the observed staining

The antibody binding specificity was assessed for all positive cases using co-localization and immunoadsorption experiments. Co-localization assays confirmed signal overlap between patient serum and commercial antibodies for MX1 and DBNL, suggesting target specificity (Fig. 3). Incubation of transfected cells with a mix of secondary antibodies without primary antibodies showed no binding.

Immunoadsorption assays showed complete signal elimination in 3 of 5 cases and significant signal reduction in 2 others after incubation with transfected cells, but no signal reduction using untransfected cells. Additional data on immunoadsorption assay results are shown in supplementary Table 1 and supplementary Fig. 2.

### Summary of clinical features of seropositive cases

#### MX1 positive cases

Three elderly individuals (2 M, 1 F, median age 79 years) were diagnosed with iSFN following the onset of sensory disturbances. All experienced paresthesias and burning pain, predominantly affecting the limbs and trunk, with symptoms ranging from subacute to chronic in onset. Pruritus was a prominent symptom in two cases. One patient complained of “cramp-like” pain and “burning mouth”. Two patients experienced episodic worsening of pain symptoms. The combination of paraesthesia, burning pain with episodic worsening and pruritus, was identified only in three further cases among the two cohorts of 244 (1.2%) SFN patients. Interestingly, these cases (2 F, 1 M) were all diagnosed with iSFN, in one case triggered by COVID-19 infection and in another by vaccination, and were much younger than MX1-IgG positive cases (median age 41 years).

Skin biopsies confirmed somatic small fiber loss, with one case also showing autonomic involvement.

Autoimmune and paraneoplastic antibody panels, including CASPR2, LGI1, and onconeural markers, were negative. MX1-IgG antibodies were detected in all three cases, with titers ranging from low to very high. The IgG1 subclass was predominant, with additional IgG4 and IgG2 present in one case (Table 3; supplementary Table 2 and supplementary Fig. 3). Only one patient, with subacute onset, received a short course of corticosteroids, with partial symptom improvement; the others were not treated with immunotherapy.

**Table 3:** Summary of results of positive cases for novel targets by CBA

Ab Target	Age (y)	Disease duration at sampling (m)	IENFD (fibers/mm)		SFN type	SFN pattern	End-point titer	IgG subclasses	Diagnosis	Immunotherapy, Outcome
			Thigh	Leg						
Main Cohort										
MX1	79	24	6.6	1.6	Somatic and autonomic	LD	1:40,960	IgG1 > IgG4 > IgG2	iSFN	NA
MX1	86	24	7.5	4.9	Somatic	NLD	1:160	IgG1	iSFN	Steroid, Improvement
DBNL	45	12	9.1	5.7	Somatic and autonomic	NLD	1:320	IgG1	AI sSFN	NA
DBNL	28	12	9.5	7.2	Somatic and autonomic	NLD	1:5120	IgG2 > IgG1	iSFN	NA
Independent replication cohort										
MX1	78	18	6.1	5.8	Somatic	NLD	1:640	IgG1	iSFN	NA

*AI* autoimmune, *F* Female, *iSFN* idiopathic small fiber neuropathy, *LD* length-dependent, *M* Male, *NA*, not available, *NLD* non-length-dependent, *sSFN* secondary small fiber neuropathy

#### DBNL positive cases

Two patients (1 M, 1 F), with a diagnosis of SFN, showed the presence of DBNL antibodies. One, experienced paresthesias, myalgias and dysautonomic symptoms and was diagnosed with sSFN due to a concomitant rheumatologic condition. The second developed subacute onset trunk and limb burning paresthesias and altered thermal perception, about two weeks after a COVID-19 vaccine. In both cases, skin biopsy confirmed somatic and autonomic small fiber loss with a NLD pattern.

Serological testing for common neuropathy-related antibodies was negative. DBNL antibodies were present in both cases, with subclass analysis showing IgG1 restriction in one, and IgG2 predominance in the other (Table 3; supplementary Table 2 and supplementary Fig. 3). Neither patient received immunotherapy.

## Discussion

Although several medical conditions can cause SFN, over 50% of cases remain idiopathic (iSFN) [23], significantly limiting the possibility of targeted therapeutic strategies.

In this study, we investigated the frequency and potential clinical relevance of autoantibodies against MX1, DBNL, and KRT8 in a large, well-characterized cohort of patients with suspected SFN. Using *in-house* fixed CBAs, we identified rare but specific seropositivity for MX1 and DBNL antibodies in SFN patients, particularly in those with idiopathic disease. These findings were corroborated in a second independent iSFN cohort, where prevalence remained low but consistent. Moreover, antibodies against either target were not found in a relatively large cohort of disease controls, further supporting the specificity of our findings.

In our main cohort, MX1 antibodies were detected in 1.7% of iSFN patients, a proportion that rose to 4.5% when excluding cases with potential immune triggers such as COVID-19 infection or vaccination. These results were confirmed in a second cohort of 31 iSFN patients, where we identified one additional MX1-IgG positive case. The frequency of MX1 positivity in the independent replication cohort was 3.2%, comparable to the 4.5% observed in the main cohort when excluding post-COVID cases. These findings reinforce the association of MX1 antibodies with a distinct subgroup of iSFN patients.

Despite the low frequency, MX1-seropositive patients shared notable clinical features, including elderly age, diffuse burning pain with episodic worsening, and pruritus. The presence of pruritus is particularly interesting, as itch has been reported as a common feature in SFN [24], though its mechanisms have not been fully elucidated [25]. Although MX1 has not been directly implicated in itch signalling, some evidence may suggest a possible indirect role in the development of itch. MX1 is an interferon-induced GTP-binding intracellular protein expressed in primary sensory neurons [26–28], where it has been proposed to interact with transient receptor potential canonical channel s (TRPC) family members, such as TRPC3, TRPC4, and TRPC5, that have been associated with acute and chronic itch and with involvement in pain plasticity [29–32]. In particular, the MX1-TRPC6 interaction has been suggested to modulate calcium influx into neurons, potentially contributing to neuronal hyperexcitability and pain [33]. Although recent studies question the specificity of MX1-TRPC6 interactions[14], the co-expression of MX1 and TRP channels in dorsal root ganglia [34, 35] and their link to peripheral sensitization via proinflammatory mediators[32] provide a plausible framework for indirect modulation of sensory neuron excitability. While no studies have demonstrated a direct mechanistic link, the presence of pruritus only in MX1-IgG positive patients among seropositive cases suggests that MX1 autoimmunity may mark a specific inflammatory or neuroimmune phenotype prone to itch. This association warrants further mechanistic investigation.

MX1-IgG belonged mostly to the IgG1 subclass, in line with a recent study [14] showing elevated MX1-IgG levels, with only IgG1, in a cohort of 59 patients with definite SFN. Using an ELISA test and confirming the specificity by CBA and flow cytometry, the Authors reported higher levels of MX1-Abs in a defined SFN patient group, and a subgroup analysis highlighted a predominance in patients with autoimmune SFN, supporting its diagnostic relevance as a biomarker [14]. These findings differ from ours, as we observed a lower frequency in our large cohort and found MX1-IgG positivity in idiopathic rather than autoimmune SFN. Although patients’ ethnicity (Asian vs Caucasian) might explain some of this discrepancy, methodological differences in detection methods, reporting metrics, and CBA protocols should also be considered. ELISA presents inherent limitations, including loss of native protein conformation and the occurrence of nonspecific antibody binding, defined as a specific serum background. In contrast, CBA maintains the native conformation of the protein and thus represents a valuable alternative for autoantibody detection. Despite the similar use of a fixed CBA protocol, inter-laboratory differences in the plasmids used, cut-off values and interpretation may lead to inconsistent findings, highlighting the need for inter-laboratory standardization.

In addition to MX1, we identified two cases of DBNL antibody positivity. DBNL is an adapter protein involved in endocytosis, cytoskeleton reorganization, neurite and synapse formation, and neuron morphogenesis [36, 37], with cytoplasmic expression in various tissues, mainly the bone marrow and lymphoid tissues [38]. Although DBNL has not been implicated in neuropathic pain, its involvement in neuronal structural integrity suggests a possible role in the pathophysiology of SFN.

Both DBNL-IgG-positive cases showed combined somatic and autonomic nerve fiber loss. Their autoantibody subclass profiles included IgG1, with one showing IgG2 dominance. Only one case had a diagnosis of iSFN, and symptoms began after COVID-19 vaccination, making DBNL an unlikely biomarker of iSFN, as previously shown by Chan et al.[13]. No DBNL positive cases were identified in the independent replication cohort.

Finally, KRT8, a cytoskeletal protein previously associated with chronic inflammatory demyelinating polyneuropathy (CIDP) and neuropathic pain[39, 40], showed no reactivity in our cohort, suggesting minimal relevance to SFN and supporting the specificity of our findings for MX1 and DBNL antibodies in this context.

Nevertheless, the intracellular localization of these two targets raises questions about their pathogenicity [19], as autoantibodies directed against intracellular targets are generally considered less likely to be directly pathogenic. Indeed, the pathogenicity of autoantibodies is predominantly associated with the cell surface location of their target, supported by the reversible effects of the Abs in both in vitro and in vivo models [41], and the good response to immunotherapy, compared to Abs against intracellular antigens [42, 43]. On the other hand, these antibodies may still play a role in immune activation or reflect disease-specific immune responses, thereby serving as potential biomarkers [44]. Notably, the only autoantibody-positive patient who received immunotherapy reported clinical improvement, suggesting potential prognostic or therapeutic implications that warrant further assessment.

Finally, given the association between COVID-19 infection and vaccination [17, 45] and SFN [7, 17], and the high proportion of patients in our cohort (40.7%) reporting symptoms in this context, we also examined the association between these triggers and antibody positivity. Only one patient, DBNL-IgG positive, developed symptoms after vaccination, providing no evidence of a direct link between COVID-19 immune triggers and the autoantibodies investigated in this study.

This study has several limitations. First, the low frequency of MX1 and DBNL autoantibodies limits the power to identify clear clinical associations with the seropositive status. Second, while co-localization and immunoadsorption supported antibody specificity, functional assays to assess pathogenicity were not performed. Third, we did not use a second confirmatory assay, and the size of the independent replication cohort was small, limiting the generalizability of our findings. Prospective studies with larger, population-based cohorts and functional characterization of the identified antibodies are needed to clarify their clinical significance.

In conclusion, we identified rare but specific MX1 and DBNL autoantibodies in a subset of patients with iSFN. MX1 antibodies, primarily IgG1, appear to define a small but clinically distinctive subgroup characterized by pruritus and diffuse neuropathic pain with episodic worsening. It is nevertheless worth noting that the small percentage of positive patients identified is insufficient to confirm a characteristic clinical phenotype, although it is intriguing and warrants further investigation by identifying additional positive cases.

Although current evidence does not support routine clinical testing, MX1-IgG may represent a candidate biomarker for a particular iSFN phenotype. Larger multicenter studies, functional analyses, and longitudinal follow-up are needed to clarify the diagnostic, prognostic, and mechanistic significance of these rare autoantibodies and to determine whether they contribute to SFN pathogenesis or reflect broader autoimmune processes within idiopathic forms of the disease.

## Supplementary Information

Below is the link to the electronic supplementary material.

Supplementary file1 (PDF 393 KB)

## Data Availability

Anonymized data not published within this article will be made available by request from any qualified investigator.
