# Supplementary material for: Autoantibodies in small fiber neuropathy: frequency and clinical features associated with antibodies to the novel targets MX1 and DBNL
Source: J Neurol. 2026 Jul 17;273(8):468. doi: 10.1007/s00415-026-13995-8 (PMC13379470; doi:10.1007/s00415-026-13995-8)
Supplement: Supplementary file 1 — Supplementary file1 (PDF 393 KB) [file 415_2026_13995_MOESM1_ESM.pdf]

Supplementary Information (SI)

Autoantibodies in Small Fiber Neuropathy: frequency and clinical features associated with antibodies to the novel targets MX1 and DBNL

Running title: MX1 and DBNL autoantibodies in SFN

Journal of Neurology

Luana Morelli[1], Fortuna Ricciardiello[1], Alessandro Furia[2,3], Alex Incensi[1], Stefano Vozzi[2], Lucrezia Serra[1], Ilaria Gligora[2], Veria Vacchiano[3], Giovanni Rizzo[3], Vincenzo Donadio[1,2,3], Rocco Liguori[1,2,3], Maria Pia Giannoccaro[1,2,3]

1 IRCCS Istituto delle Scienze Neurologiche di Bologna, Programma di Patologia Neuromuscolare e Neuroimmunologia, Bologna, Italy

2 Dipartimento di Scienze Biomediche e Neuromotorie, Università di Bologna, Bologna, Italy

3 IRCCS Istituto delle Scienze Neurologiche di Bologna, UOC Clinica Neurologica, Bologna, Italy

Corresponding Author: Maria Pia Giannoccaro, IRCCS Istituto delle Scienze Neurologiche di Bologna e Dipartimento di Scienze Biomediche e Neuromotorie, Università di Bologna, Via Altura 3, 40139 Bologna, Italy, mariapia.giannoccar2@unibo.it

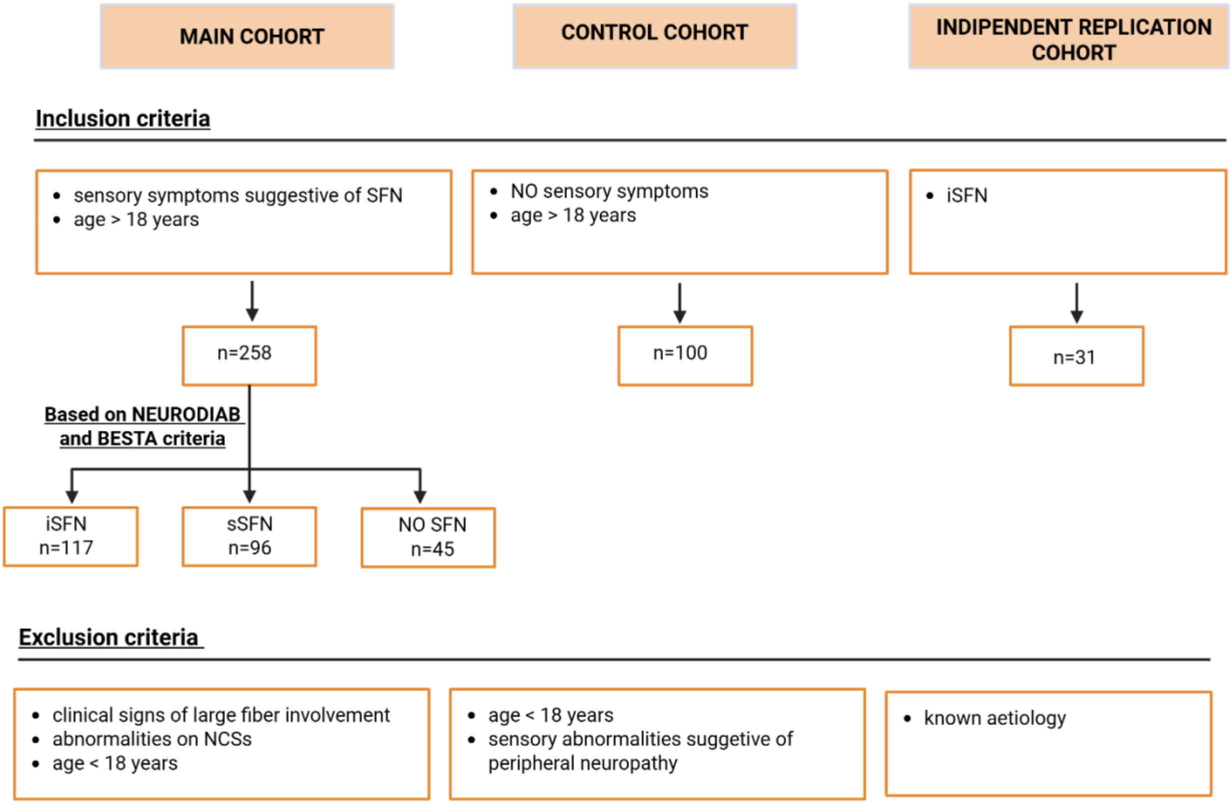

Figure S. 1 - Flow chart of the cohort study design

Selection of the cohorts with exclusion and inclusion criteria.

Table S. 1 - Summary of immunoadsorption assay results of positive cases

Ab Target	Case number	Initial dilution	Number of immunoadsorption steps	Total absorption time (hs)
**Main Cohort**				
**MX1**	Case 1	1:640*	8	9 **
**MX1**	Case 2	1:20	5	6
**DBNL**	Case 1	1:20	2	3
**DBNL**	Case 2	1:200*	7	8
**Independent replication cohort**				
**MX1**	Case 3	1:20	4	5

Results of the immunoabsorption assay for positive cases: number of absorption steps and incubation time. *For high-titre sera, a higher starting dilution was used based on prior testing, to avoid excessive dilution steps and potential sample contamination affecting assay reliability

**After 7hs, the score remains 0,5/1

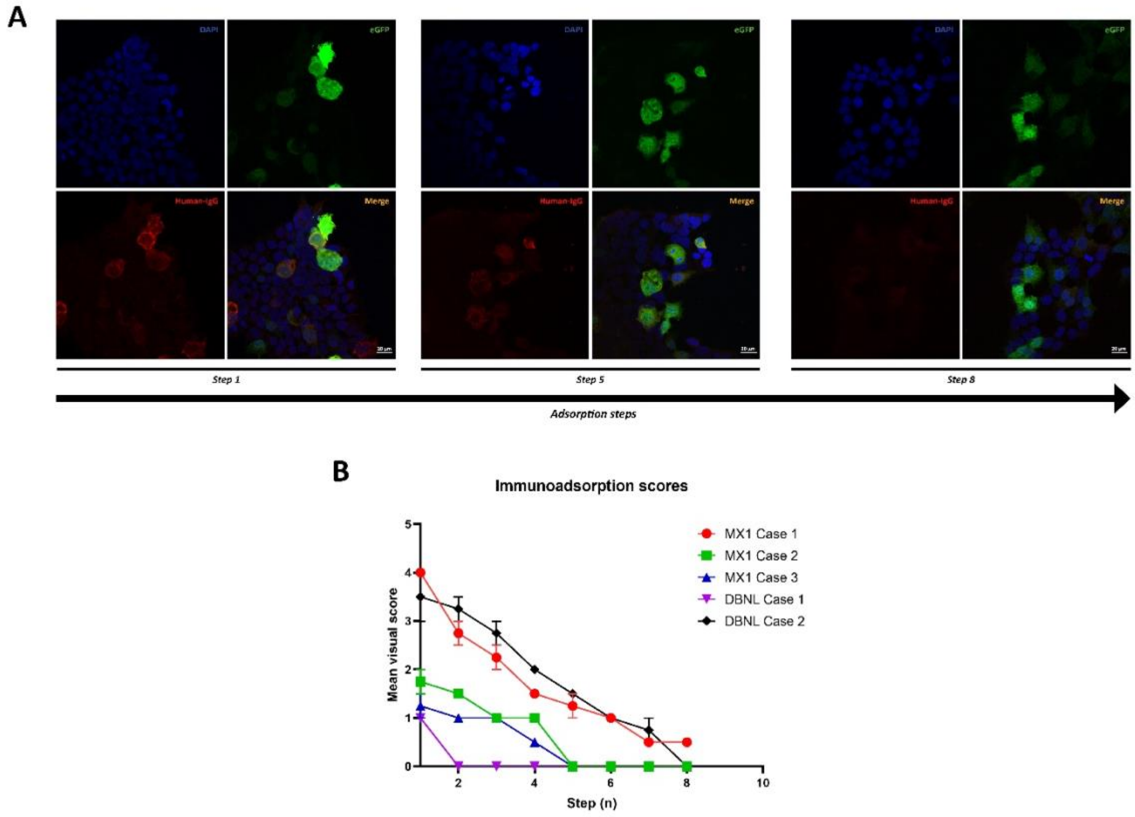

Figure S. 2 - Immunoadsorption assay: immunoadsorption scores at the different steps of positive cases and staining pattern in the serum of DBNL positive case 2

(A) Representative confocal images showing the decrease in immunofluorescence signal intensity in the serum of DBNL positive case 2 in the immunoabsorption assay. Scale bar: 20 µm; (B) Trend observed in sera from positive cases in the immunoabsorption assay. For graphical representation, visual scores were approximated upwards. Data show the mean of two immunoabsorption assays performed for each serum sample and standard error (SEM). Sera were tested at a 1:20 dilution, except for high-titer samples (MX1 Case 1 and DBNL Case 2; starting dilutions 1:640 and 1:200, respectively.

Table S. 2 - Summary of IgG subclasses of positive cases

Ab Target	Case number	IgG subclasses	Endpoint dilution
**Main Cohort**			
**MX1**	Case 1	IgG1>IgG4>IgG2	1:20480 (IgG1)
			1:2560 (IgG2)
			1:10240 (IgG4)
**MX1**	Case 2	IgG1	320
**DBNL**	Case 1	IgG1	1:80
**DBNL**	Case 2	IgG2>IgG1	1:640 (IgG1)
			1:2560 (IgG2)
**Independent replication cohort**			
**MX1**	Case 3	IgG1	1:80

Summary of IgG subclass characterization in sera from positive cases, showing the distribution of specific IgG subclasses and their corresponding endpoint titres.

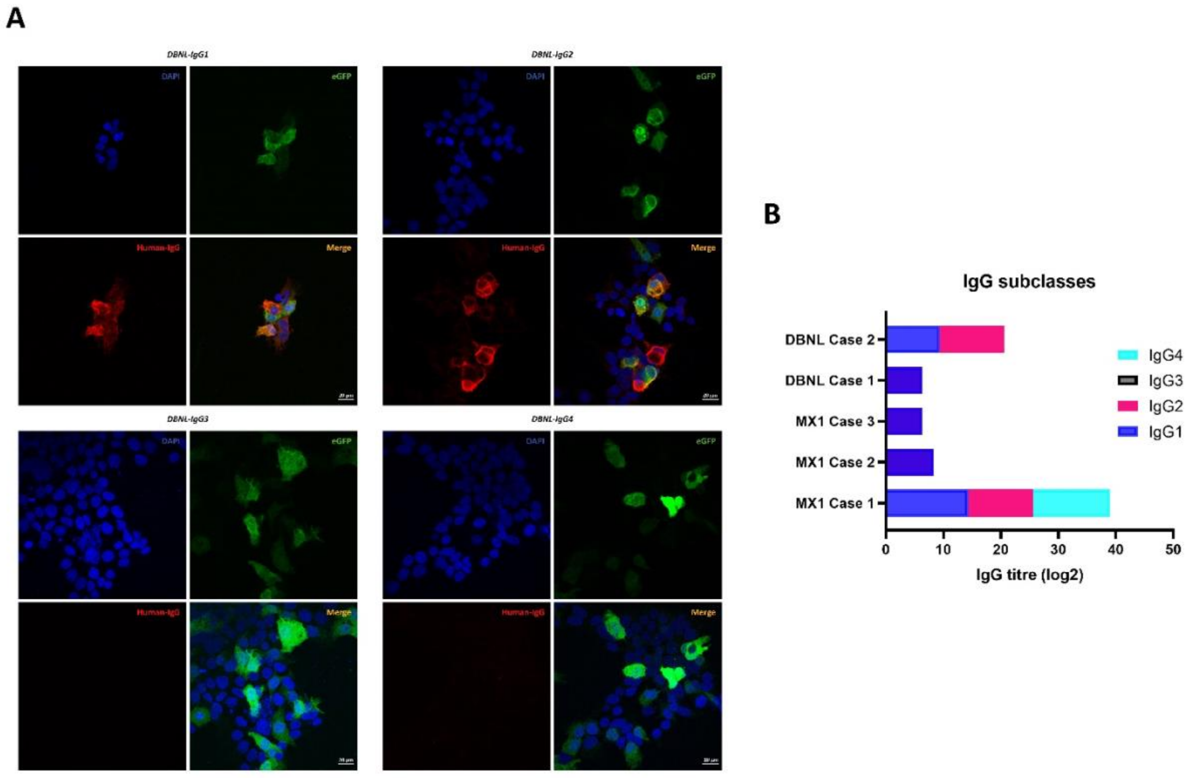

Figure S. 3 - Characterisation of IgG subclasses in the sera of positive cases

(A) Representative confocal images showing IgG subclasses in the serum of DBNL positive case 2. Scale bar: 20 µm; (B) Distribution of IgG subclasses in sera from positive cases.
